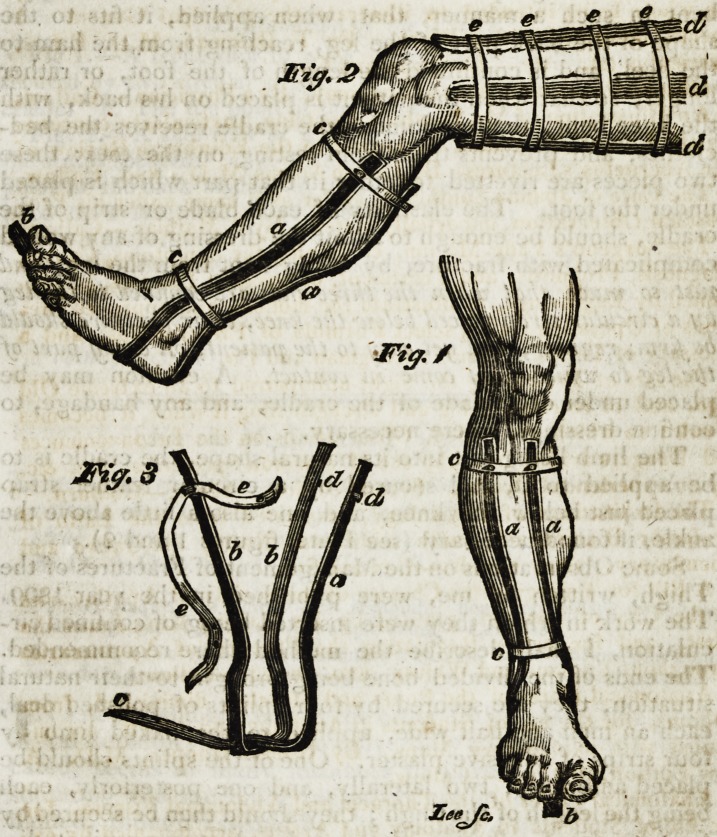# Remarks on Fractures of the Extremities, with the Description of an Elastic Iron Cradle for Fractures of the Leg

**Published:** 1815-03

**Authors:** Francis Bush

**Affiliations:** Surgeon.


### Medical and Physical Journal.

3 OF VOL. XXXIII.] MARCH, 1815. [NO. 193.

*For the Medical and Physical Journal.*

### *Remarks on Fractures of the Extremities, with the Description of an Elastic Iron Cradle for Fractures of the Leg; by* FRANCIS BUSH, Surgeon.

AS the result of many practical trials, I feel confidence in recommending the method now suggested of confining fractures of the extremities, especially those of the leg and thigh, as better calculated to prevent deformity of the parts injured: at the same time it occasions less pain to the patient than than any hitherto taught in the schools of surgery, or by writers on that subject.

I might never have paid so much attention to the treatment of such case, but for an accident which occurred on my own person. A horse having tumbled with me and fractured my leg, rendered the method of confining the bones, with the greatest certainty and ease, a matter peculiarly interesting to me; and led to the use of the Elastic Iron Cradle (see Plate, fig. 3d). It is formed by two pieces of thin hoop iron, from half an inch to an inch wide; one of them is bent like a stirrup under the foot, and over each ankle, reaching up to the knee on both sides; the other is bent in such a manner, that, when applied, it fits to the shape of the back part of the leg, reaching from the ham to the heel, and is continued the length of the foot, or rather longer, so that when the patient is placed on his back, with the knee bent, the foot joint of the cradle receives the bedclothes, and prevents them from resting on the toes: these two pieces are rivetted together in that part which is placed under the foot. The elasticity of each blade or strip of the cradle, should be enough to admit the dressing of any wound complicated with fracture, by being bent from the leg, *and just so much that when the three ends are confined to the leg by a circular strap placed below the knee, their pressure should be firm, regular, and yet easy to the patient, on every part of the leg to which they come in contact.* A cushion may be placed under each blade of the cradle, and any bandage, to confine dressings, where necessary.

The limb being put into its natural shape, the cradle is to be applied to it, and secured by a circular leather strap placed just below the knee, and one also a little above the ankle, if found necessary (see Plate, figures 1 and 2).

Some Observations on the Management of Fractures of the Thigh, written by me, were published in the year 1809. The work in which they were inserted being of confined circulation, I shall describe the method there recommended. The ends of the divided bone being brought to their natural situation, they are secured by four splints of polished deal, each an inch and half wide, applied to the naked limb by four strips of adhesive plaster. One of the splints should be placed anteriorly, two laterally, and one posteriorly, each being the length of the thigh; they should then be secured by four bands of adhesive plaster, placed at convenient distances (see plate, fig. 2); the bands may be loosened or tightened, as may be necessary. I have treated many cases in this manner, where the swelling has not required their being loosened or removed during the cure. The patient should be placed on his back, with the knee bent, in cases of fractured thighs and legs.

In fractures of the upper and lower arm, I use splints similar to those for the thigh, which are likewise confined by strips of adhesive plaster.

Injuries of this kind near the joints will sometimes be productive of stiffness, but, by a proper application of the means I have detailed, a distorted limb from a fracture will be a rare occurrence. This cradle will be a very convenient instrument on ship-board, and where it is necessary to remove the patient to a distance from where the accident happens.

*Explanation of the Plates.*

Fig 1.—A front view of a leg placed in the elastic iron cradle, secured by two circular straps. *a, a.* The two side blades of the cradle. *b.* The toe point of the hind blade. *c, c.* Circular straps.

Fig. 2.—A side view of a leg placed in the elastic cradle, with the straps secured. *a, a.* The back and side blades. *b.* The toe point of the back blade. *d, d, d.* Three of the deal splints applied to a fractured thigh, secured by four adhesive circular bands, *e, e, e, e.*

Fig. 3.—The elastic iron cradle when off the leg. *a.* Hind blade. *b, b.* Side blades. *c.* Toe point. *d, d.* Studs to fix the strap. *e, e.* The strap.

*Frome, Dec.* 30, 1814. FRANCIS BUSH.

## Figures and Tables

**Fig. 2 Fig. 1 Fig. 3 f1:**